# Middle cerebral artery occlusion due to cardiac tissue following catheter ablation in Wolff–Parkinson–White syndrome

**DOI:** 10.1093/jscr/rjab269

**Published:** 2021-06-29

**Authors:** Subash Phuyal, Raju Paudel, Pooja Agrawal, Ritesh Lamsal

**Affiliations:** Department of Neuroimaging and Interventional Neuroradiology, Grande International Hospital, Kathmandu, Nepal; Department of Neurology, Grande International Hospital, Kathmandu, Nepal; Department of Radiology, Norvic International Hospital, Kathmandu, Nepal; Department of Anaesthesiology, Tribhuvan University Teaching Hospital, Kathmandu, Nepal

## Abstract

Catheter ablation is a commonly performed procedure in patients with Wolff–Parkinson–White syndrome. A 56-year-old man developed an acute ischemic stroke immediately after undergoing radiofrequency catheter ablation of the left-sided accessory pathway. Neuroimaging revealed complete occlusion of the proximal middle cerebral artery. Mechanical thrombectomy (MT) was performed with successful retrieval of the thrombo-embolus. Histopathological examination of the thrombo-embolus confirmed organic cardiac tissue. The patient was later discharged from the hospital with no neurologic deficit. There is no report of successful MT in patients with large-vessel occlusion because of the embolization of cardiac tissue after catheter ablation. This report highlights the need to remain vigilant for signs of stroke after any cardiac intervention.

## INTRODUCTION

There are several indications of catheter ablation in patients with Wolff–Parkinson–White (WPW) syndrome. During the procedure, intra-cardiac clots, cardiac atheroma, eschar, tumor tissue and air bubbles can sometimes cause cardio-embolic stroke. However, the reported incidence of stroke after catheter ablation is low. Large-vessel occlusion (LVO) stroke with organic cardiac tissue is rarely reported. We report successfully performing mechanical thrombectomy (MT) in a patient with proximal middle cerebral artery (MCA) occlusion with complete recanalization after the procedure. To the best of our knowledge, the successful retrieval of organic cardiac tissue in a patient with LVO stroke immediately after undergoing catheter ablation has not been previously reported.

## CASE REPORT

**Figure 1:**
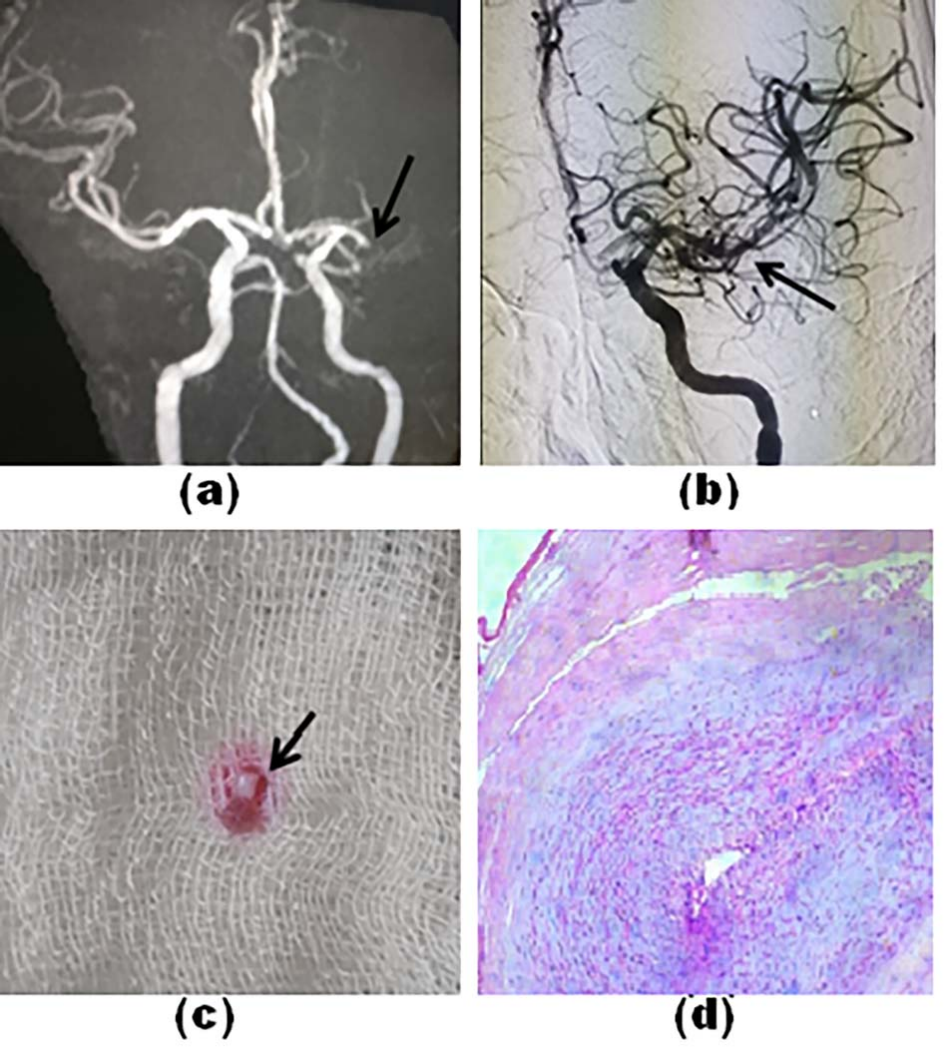
Time-of-flight MR angiography shows a complete cut-off of left M_1_-MCA (**a**, arrow). The final angiogram shows complete recanalization of the MCA and its branches (**b**, arrow). The tissue thrombus resembled an organic tissue (**c**). Histopathology of the tissue thrombus shows parallel bundles of muscle fiber (**d**).

A 56-year-old man developed acute-onset right-sided weakness and slurring of speech after undergoing radiofrequency catheter ablation of the left-sided anterolateral accessory pathway. Magnetic resonance imaging (MRI) showed restricted diffusion in the left MCA territory (MR-Alberta Stroke Program Early CT Score: 8). An MR angiogram showed complete occlusion of the left proximal MCA (Fig. 1a). We performed MT using the a direct aspiration first pass technique (ADAPT) technique with complete recanalization after a single pass (Fig. 1b). The retrieved thrombo-embolus resembled an organic tissue (Fig. 1c). Histopathological examination of the tissue thrombus found close parallel bundles of muscle fibers, suggestive of cardiac tissue (Fig. 1d). At the time of hospital discharge, the patient did not have any neurologic deficit.

## DISCUSSION

Cardio-embolic strokes account for nearly one-fifth of acute strokes and have one of the highest mortality among all stroke subtypes [1]. Most cardio-embolic strokes result from underlying heart conditions that promote blood stasis, such as atrial fibrillation, myocardial infarction and dilated cardiomyopathy.

Catheter ablation in WPW syndrome reportedly carries a low risk of cardio-embolic stroke [2]. Proposed explanations for embolization include dislodgement of a pre-existing clot, development of thrombi within the catheter, char formation at the ablation site, air embolism and accidental trauma to normal cardiac tissue, such as the valve, annulus or sub-valvular structures [2]. However, the true incidence of strokes after catheter ablation is difficult to determine as most episodes are silent infarcts [2]. Studies show that the adoption of better peri-procedural anticoagulation strategies has resulted in a steady decline in cardio-embolic strokes after catheter ablation [3]. Nevertheless, these anticoagulation strategies may not decrease the risk of stroke caused by organic cardiac tissue embolus. Even a small embolus can cause an LVO as catheter ablation leads to immediate systemic activation of platelets and the coagulation cascade [4]. Surprisingly, there is a paucity of information about strokes caused by cardiac tissue embolization, and the incidence is probably higher in more invasive interventions. In our case, histopathology could not locate the exact cardiac site of the embolus, but it likely originated from the papillary muscle–chordae tendineae complex, akin to a previous report where chordae tendineae caused LVO stroke after valve surgery [5].

Physicians need to be watchful for signs of stroke after cardiac interventions. As thrombolysis is not useful in stroke caused by organic thrombi, neurointervention facilities are valuable in centers that perform cardiac interventions. In our knowledge, this is the first report that describes MT in LVO stroke from a cardiac tissue embolus following catheter ablation.

## CONFLICT OF INTEREST STATEMENT

None declared.

## FUNDING

None.
